# Measurement of Mandibular Growth Using Cone-Beam Computed Tomography: A Miniature Pig Model Study

**DOI:** 10.1371/journal.pone.0096540

**Published:** 2014-05-06

**Authors:** Hsien-Shu Lin, Yunn-Jy Chen, Jia-Da Li, Tung-Wu Lu, Hau-Hung Chang, Chih-Chung Hu

**Affiliations:** 1 School of Dentistry, National Taiwan University, Taipei City, Taiwan, R.O.C.; 2 Institute of Biomedical Engineering, National Taiwan University, Taipei City, Taiwan, R.O.C.; 3 Department of Orthopaedic Surgery, School of Medicine, National Taiwan University, Taipei City, Taiwan, R.O.C.; 4 Department of Mechanical Engineering, Ming-Chih University of Science and Technology, New Taipei, Taiwan, R.O.C.; Mayo Clinic College of Medicine, United States of America

## Abstract

The purpose of this study was to measure the long-term growth of the mandible in miniature pigs using 3D Cone-Beam Computerized Tomography (CBCT). The mandibles of the pigs were scanned monthly over 12 months using CBCT and the 3D mandibular models were reconstructed from the data. Seventeen anatomical landmarks were identified and classified into four groups of line segments, namely anteroposterior, superoinferior, mediolateral and anteroinferior. The inter-marker distances, inter-segmental angles, volume, monthly distance changes and percentage of changes were calculated to describe mandibular growth. The total changes of inter-marker distances were normalized to the initial values. All inter-marker distances increased over time, with the greatest mean normalized total changes in the superoinferior and anteroposterior groups (p<0.05). Monthly distance changes were greatest during the first four months and then reduced over time. Percentages of inter-marker distance changes were similar among the groups, reaching half of the overall growth around the 4^th^ month. The mandibular volume growth increased non-linearly with time, accelerating during the first five months and slowing during the remaining months. The growth of the mandible was found to be anisotropic and non-homogeneous within the bone and non-linear over time, with faster growth in the ramus than in the body. These growth patterns appeared to be related to the development of the dentition, providing necessary space for the teeth to grow upward for occlusion and for the posterior teeth to erupt.

## Introduction

The growth and the associated morphological changes of the mandible has long been an interesting research subject for orthodontists [1]. Before the maturation of the mandible, any orthodontic treatment or orthognathic surgery is likely to involve variables related to the growth patterns. Therefore, if treatments are needed during the growth period, the factor of the mandibular growth should be taken into account.

The growth of the mandible in humans was first studied by Bjork in 1955 [2] using lateral cephalometric radiographs. Metallic pins were implanted into the mandible as landmarks for monitoring bone growth. While useful baseline information was provided by this and subsequent studies [3], [4], the data were limited to two-dimensional (2D) measurements. In contrast to cephalometric radiographs, computed tomography (CT) can accurately measure the three-dimensional (3D) geometry of bones, but requires relatively high doses of radiation, limiting its use in *in vivo* studies. In recent years, the development of cone-beam CT (CBCT) has enabled the accurate measurement of the 3D geometry of bones at relatively low radiation levels. This has opened up the possibility of long-term monitoring of the mandibular growth on animal models. Kim *et al*. [5] studied mandibular growth in New Zealand white rabbits using CBCT, but measurements were performed with relatively large time intervals with a limited number of variables for describing the bone geometry. The fundamental differences in the gross morphology between the mandibles of rabbits and humans [6] are also a major concern. On the other hand, owing to the radiation dosage, using CT for human mandibular growth studies is still unacceptable, except in special cases where a routine CT scan is necessary, such as in children with Apert syndrome [7]. However, since the mandibles in these children are CT scanned at a limited number of instances unevenly spread over a period of time (about five times over 14 years), the descriptions of the patterns of mandible growth in this particular group of subjects are limited to the selected time instances.

Despite the importance of long-term monitoring of the mandibular growth at well-defined time intervals, data of the mandibular growth in humans is not currently available because long-term accurate tracking of the mandibular growth in three dimensions on a regular basis with constant time intervals remains difficult, if not impossible, to achieve. Given the current limitations in human studies due to ethical considerations, animal models remain a viable alternative to human subjects. Miniature pigs are good alternative to humans in mandibular growth studies because they are similar to humans in terms of the morphology of the mandible (size and shape), the ways of chewing and grinding, and their bone metabolism rate [8]–[10].

The purpose of this study was to measure the long-term, 3D morphological changes of the mandible in miniature pigs during growth using CBCT in order to provide baseline data of the mandibular growth for future scientific and clinical applications. It was hypothesized that the growth of the mandible would be anisotropic and non-homogeneous within the bone and non-linear over time.

## Materials and Methods

**Figure 1:**
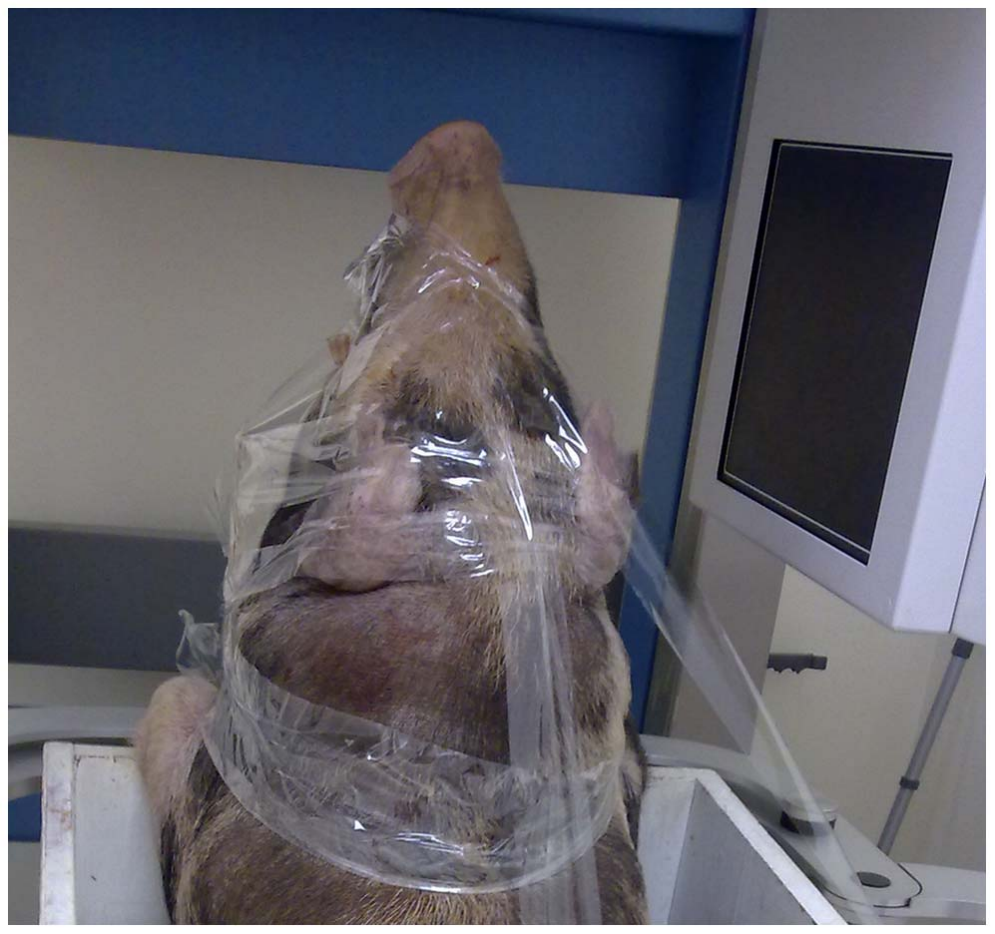
The experimental setup for the CBCT scan of a typical subject.

Eight Lee-Sung strain miniature pigs raised on a certified farm for experimental animals (temperature: 26 to 28°C; humidity: 55 to 60%) were used in the current study. An *a priori* power analysis based on pilot results using GPOWER [11] determined that four miniature pigs would yield a power of 0.8 at a significance level of 0.05. From the age of one month onwards, each of the pigs was given a CT scan of the mandible once every four weeks over a period of a year. To avoid differences in the number of days between calendar months, and to simplify the description of the changes over the time intervals, a time interval of four weeks was referred to as a ‘month’ (T =  time interval  = 4 weeks), so a total of twelve sets of CT data were obtained for each pig (T = T1, T2, …, T12). At each CT scan, the pigs were under general anesthesia by an intramuscular injection of 1 cc/10 kg of zoletil 50 (50 mg/kg) (Virbac Laboratories, Carros, France). To prevent choking, saliva production was inhibited by an intramuscular injection of atropine sulfate (Antopin, 1 mg/ml; 0.5 cc: <20 kg; 1 cc: >20 kg; Sinton Chem & Pharm Co. Ltd, Taiwan). The CT scanning was performed using a low radiation dose CBCT system (i-CAT, Imaging Sciences International, Inc., USA) with a voxel size of 0.25 mm×0.25 mm×0.25 mm and a grey intensity of 12 bits. The CBCT was chosen for the study because it provides substantial dose reductions of between 76.2% and 98.5% compared to conventional CT [12]–[14]. According to the literature, the effective dose for a maxillo-mandibular scan using conventional CT is 2100 µSv [15], while that using an i-CAT CBCT scan under the full field of view is 182.1 µSv [16]. The actual measurement of the radiation for the i-CAT CBCT scan was also made using a portable dosimeter (AT1121, ATOMTEX, Minsk, Belarus), giving an equivalent dose of 263 µSv. The CBCT system was operated with a tube potential of 1201 kVp and a tube current of 3-8 mA, and the field of view was 22 cm (height) ×16 cm (diameter) with the Extended Field of View model provided by the system. During the CBCT scan, the pig was restrained on a purpose-built workbench using transparent tape, and the mandible was positioned within the center of the region of interest with the guidance of an optical localizer in the shape of a cross (Fig. 1). This study was carried out in strict accordance with the recommendations in the *Guide for the Care and Use of Laboratory Animals* by the National Institutes of Health. The protocol was approved by the Committee on the Ethics of Animal Experiments of the National Taiwan University (Permit Number: 20080124).

**Figure 2:**
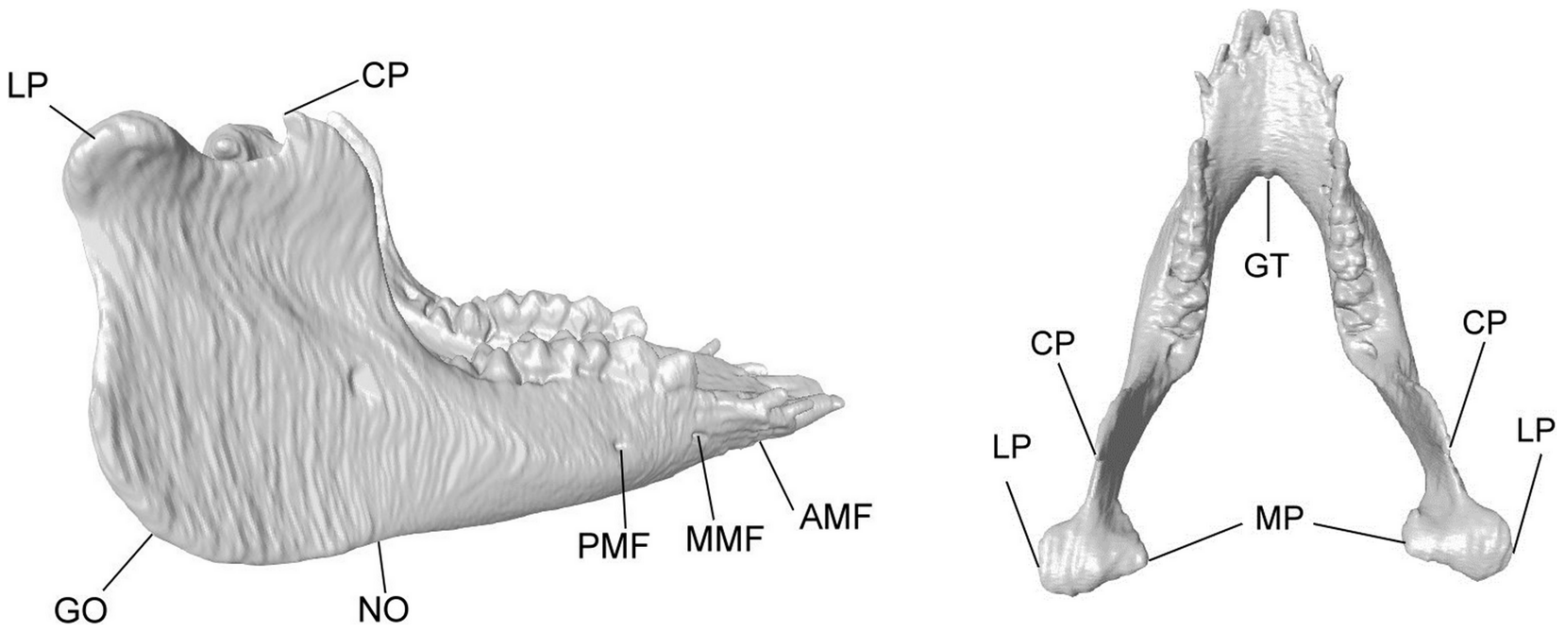
Anatomical landmarks of the mandible considered in the current study as indicated on the 3D mandible model viewed from the right and from the top.

Each of the CT data sets was used to reconstruct a 3D model of the mandible using a commercial image-processing package (Amira, Visage Imaging Inc., USA). A total of 17 anatomical landmarks on the mandible were marked on the model to describe the key morphological features of the mandible (Fig. 2 and Table 1). These anatomical bony landmarks were also selected because they were relatively easy to identify so that the repeatability of the identification of the landmarks could be maximized. Some of the bony landmarks were identified automatically by the computer to minimize human involvement, namely CP, LP, MP, GT and GO, while others were identified manually by an experienced dentist (LHS), namely AMF, MMF, PMF and NO.

**Table 1:** Anatomical landmarks on the mandible monitored during growth.

Anatomical landmark	Definition
LP: Lateral pole of condyle	The most protruding point on the lateral side of the mandibular condyle
MP: Medial pole of condyle	The most protruding point on the medial side of the mandibular condyle
CP: Coronoid process	The most protruding point on the coronoid
GO: Gonion	The most posterior and inferior point at the mandibular angle
AMF: Anterior mental foramen	The most anterior edge of the export of the mental nerve
MMF: Middle mental foramen	The middle edge of the export of the mental nerve
PMF: Posterior mental foramen	The most posterior leading edge export of the mental nerve
GT: Genial tubercle	The most prominent point of the genial tubercle
NO: Groove for external maxillary artery	The notch of the groove for the external maxillary artery

There were a total of 17 landmarks considering both sides of the mandible.

**Figure 3:**
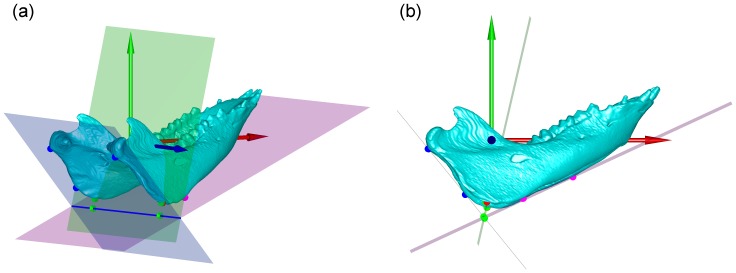
A schematic diagram illustrating the computer-based method for defining the GO point on the mandible: (a) oblique view, and (b) sagittal view. To determine GO, a plane (cyan, with cyan contact points) containing the posterior ramus borders (or tangent to the borders in mathematical terms), and a second plane (pink, with pink contact points) containing the inferior corpus borders (or tangent to the borders in mathematical terms) were established to form an obtuse angle. A third plane (green, with green contact points) passing through the intersection of the first two planes and bisecting their obtuse angle meets the curved gonial edge, and the red point on the intersected edge and closest to the intersection of the first two planes gives the GO (gonion). The axes of the local coordinate system are shown as arrows in red, blue and green.

For those landmarks identified automatically, the CPs were determined as the point of the largest curvature on the coronoid; the GT was determined on the most posterior point of the genial tubercle; the MPs were determined as the most medial point of the condyle; and the LPs were determined as the most lateral point of the condyle. Note that the gross position of the coronoid, genial tubercle and the condyles were identified manually for the subsequent accurate identification of the associated bony landmarks. The GO was determined as the point along the rounded posteroinferior corner of the mandible between the ramus and the body. To determine the point, a plane containing the posterior ramus borders (or tangent to the borders in mathematical terms), and a second plane containing the inferior corpus borders (or tangent to the borders in mathematical terms) were established to form an obtuse angle (Fig. 3). A third plane passing through the intersection of the first two planes and bisecting their obtuse angle meets the curved gonial edge, and the point on the intersected edge and closest to the intersection of the first two planes gives the GO (gonion) (Fig. 3). The auto-detected bony landmarks were verified by an experienced dentist (LHS) before being used for subsequent analysis.

The other bony landmarks, i.e., AMF, MMF, PMF and NO, were manually identified directly on the surface model in the Geomagic 3D software (Geomagic, Inc., USA) by an experienced dentist (LHS). The reliability of this procedure was determined by repeated identification of the landmarks by the same dentist, giving an Intra-Class Correlation Coefficient (ICC) of 0.9, which was considered strong for the current purpose.

Once the bony landmarks had been identified, line segments were then defined for selected pairs of markers. Distances between marker pairs or line segment lengths (*inter-marker distance*), angles between selected line segments (*inter-segmental angles*), and the *bone volume* of the mandible were calculated (Table 2). The line segments (marker pairs) were divided into four groups according to their spatial orientations, namely anteroposterior (AP), superoinferior (SI), mediolateral (ML) and anteroinferior (AI) (Table 2). Over the period of monitoring, the values of all the above-mentioned parameters, and their monthly changes, as well as the accumulated values as percentages of total changes, were calculated for each time interval using an in house-developed program in MATLAB (MathWorks, Inc., USA). The total changes of the inter-marker distances for each of the marker pairs were also normalized to their corresponding initial values (i.e., change of inter-marker distances between T12 and T1 divided by inter-marker distance at T1) to give the *Normalized Total Change*. The changes in the parameters over time represent the changes of the size of the mandible during growth. *Inter-marker distance* represent one of the dimensions of the mandible; *inter-segmental angles* represent one of the angular dimensions of the mandible; and the *bone volume* is an index for the modeling of the mandible. *Monthly changes* represent the amount of monthly growth, defined as the difference between the current and the previous month. *Percentage of change* of a parameter represents the accumulated growth at a monitoring time instance as a percentage of the *total change* of that parameter over the monitoring period (i.e., 48 weeks). Values of each of the calculated variables at each time instance of measurement were averaged across all the subjects and presented over the monitoring period. For the effect of orientation (i.e., AP, SI, ML and AI) on the normalized total changes, a one-way repeated measures analysis of variance (ANOVA) was performed. If a significant orientation effect was found, pair-wise comparisons between orientations were performed using a paired t-test with Bonferroni correction (α = 0.05/6 = 0.0082). All statistical analysis was performed using SPSS version 13.0 (SPSS Inc., Chicago, IL, USA).

**Table 2:** Inter-marker distances and inter-segmental angles measured during the monitoring period.

AP	SI	AI	ML	Angle
(anteroposterior)	(superoinferior)	(anteroinferior)	(mediolateral)	
AMF-GO	GO-CP	GT-CP	Bi-AMF	LP-GO-AMF
GO-PMF	GO-LP	GT-LP	Bi-PMF	Bi-GO-GT
NO-PMF	GO-MP	AMF-CP	Bi-CP	
MMF-PMF		GT-MP	Bi-MP	
AMF-MMF		AMF-LP	Bi-MMF	
AMF-NO		AMF-MP	Bi-LP	
CP-MP			Bi-GO	
GO-NO				
CP-LP				

Bi: Bilateral.

Definitions of the abbreviations of the markers are given in Table 1.

## Results

**Figure 4:**
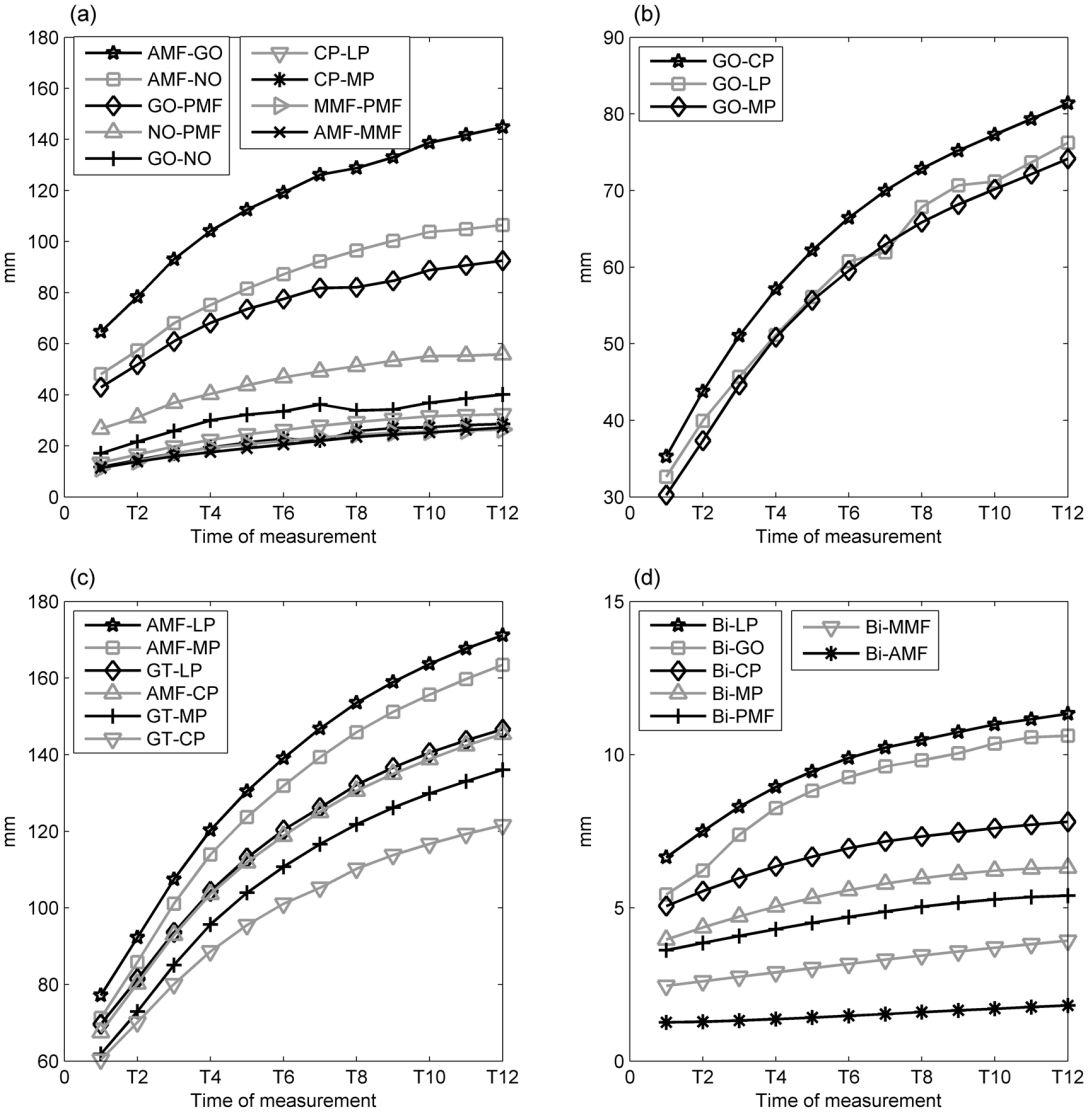
Inter-marker distances of the left side of the mandible over the monitoring period for the (a) anteroposterior, (b) superoinferior, (c) anteroinferior, and (d) mediolateral marker groups. Definitions of the abbreviations of the markers are given in Table 1.

During the monitoring period, all inter-marker distances increased over time, those for AMF-LP increasing the most and reaching a total change of 92 mm at T12, and those for BI-AMF increasing the least and reaching a total change of only 7 mm at T12 (Fig. 4). Considering the initial inter-marker distances (i.e., at T1), the mean normalized total changes of the inter-marker distances were found to be the greatest in the superoinferior group, followed by the anteroposterior and the anteroinferior groups, with those for the mediolateral group being the smallest (Table 3). In the superoinferior group, the normalized total change of the inter-marker distance for GO-MP was significantly greater than those of the other two marker pairs (GO-MP vs. GO-CP: p = 0.002; GO-MP vs. GO-LP: p = 0.002, Table 3). In the anteroposterior group, the normalized total changes in the posterior part tended to be greater than in the anterior part, but without significant differences (p = 0.083). In this group, CP-LP was the greatest and GO-PMF was the smallest with a significant difference (p = 0.011) (Table 3). In the anteroinferior group AMF-LP was the greatest, while Bi-LP and Bi-GO were the greatest in the mediolateral group (Table 3).

**Table 3:** Means (SD) of the initial values, total changes and normalized total changes of the inter-marker distances for each of the marker pairs of the mandible.

Group	Parameters	Initial Value	Total Change	Normalized Total Change	Group Mean (SD)	Between-Group P-value
AP					1.32(0.11)	P_AP-SI_ = 0.453
	Mandible				1.22(0.03)	P_AP-AI_ = 0.005*
	AMF-GO	6.46(0.49)	14.51(0.38)	1.26(0.17)		P_AP-ML_ = 0.004*
	GO-PMF	4.27(0.29)	9.30(0.23)	1.19(0.18)		P_SI-AI_<0.001*
	Anterior (Body)				1.27(0.09)	P_SI-ML_<0.001*
	NO-PMF	2.60(0.17)	5.61(0.20)	1.16(0.14)		P_AI-ML_<0.001*
	MMF-PMF	1.15(0.10)	2.61(0.35)	1.27(0.26)		
	AMF-MMF	1.15(0.15)	2.72(0.22)	1.41(0.39)		
	AMF-NO	4.77(0.35)	10.61(0.46)	1.23(0.09)^i^		
	Posterior (Ramus)				1.45(0.01)	
	CP-MP	1.20(0.12)	2.87(0.16)	1.43(0.33)		
	GO-NO	1.75(0.18)	4.24(0.40)	1.46(0.43)		
	CP-LP	1.33(0.07)	3.26(0.20)	1.47(0.25)		
SI					1.37(0.07)	
	GO-CP	3.53(0.13)	8.14(0.40)	1.31(0.12)		
	GO-LP	3.25(0.04)	7.58(0.26)	1.33(0.07)		
	GO-MP	2.99(0.05)	7.40(0.21)	1.47(0.08)		
AI					1.17(0.09)	
	GT-CP	6.06(0.29)	12.19(0.52)	1.01(0.07)		
	GT-LP	6.96(0.24)	14.69(0.18)	1.11(0.06)		
	AMF-CP	6.74(0.39)	14.52(0.71)	1.16(0.10)		
	GT-MP	6.17(0.28)	13.61(0.12)	1.21(0.09)		
	AMF-LP	7.69(0.37)	17.09(0.39)	1.23(0.09)		
	AMF-MP	7.09(0.41)	16.30(0.32)	1.30(0.12)		
ML					0.62(0.16)	
	Bi-AMF	1.25(0.11)	1.82(0.16)	0.45(0.08)		
	Bi-PMF	3.62(0.10)	5.40(0.16)	0.49(0.05)		
	Bi-CP	5.06(0.14)	7.81(0.29)	0.54(0.05)		
	Bi-MP	3.97(0.15)	6.31(0.28)	0.59(0.07)		
	Bi-MMF	2.46(0.07)	3.93(0.11)	0.60(0.06)		
	Bi-LP	6.65(0.18)	11.33(0.38)	0.71(0.06)		
	Bi-GO	5.43(0.35)	10.66(0.23)	0.97(0.12)		

The ‘group mean’ shows the mean of the normalized total changes over the whole group of markers. (unit: cm) Definitions of the abbreviations of the markers are given in Table 1.

**Figure 5:**
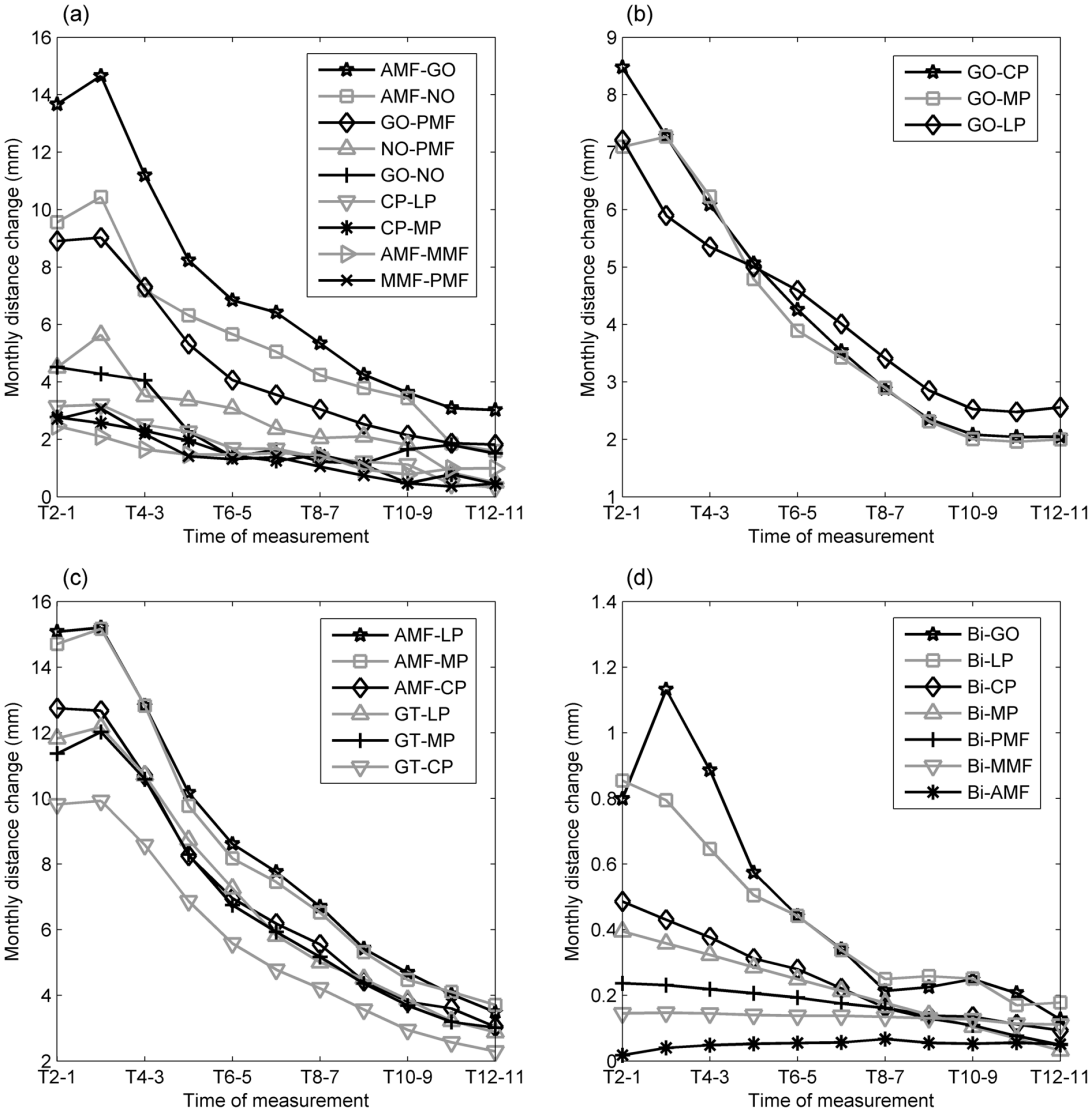
Monthly changes of the inter-marker distances of the left side of the mandible over the monitoring period for the (a) anteroposterior, (b) superoinferior, (c) anteroinferior, and (d) mediolateral marker groups. Definitions of the abbreviations of the markers are given in Table.

**Figure 6:**
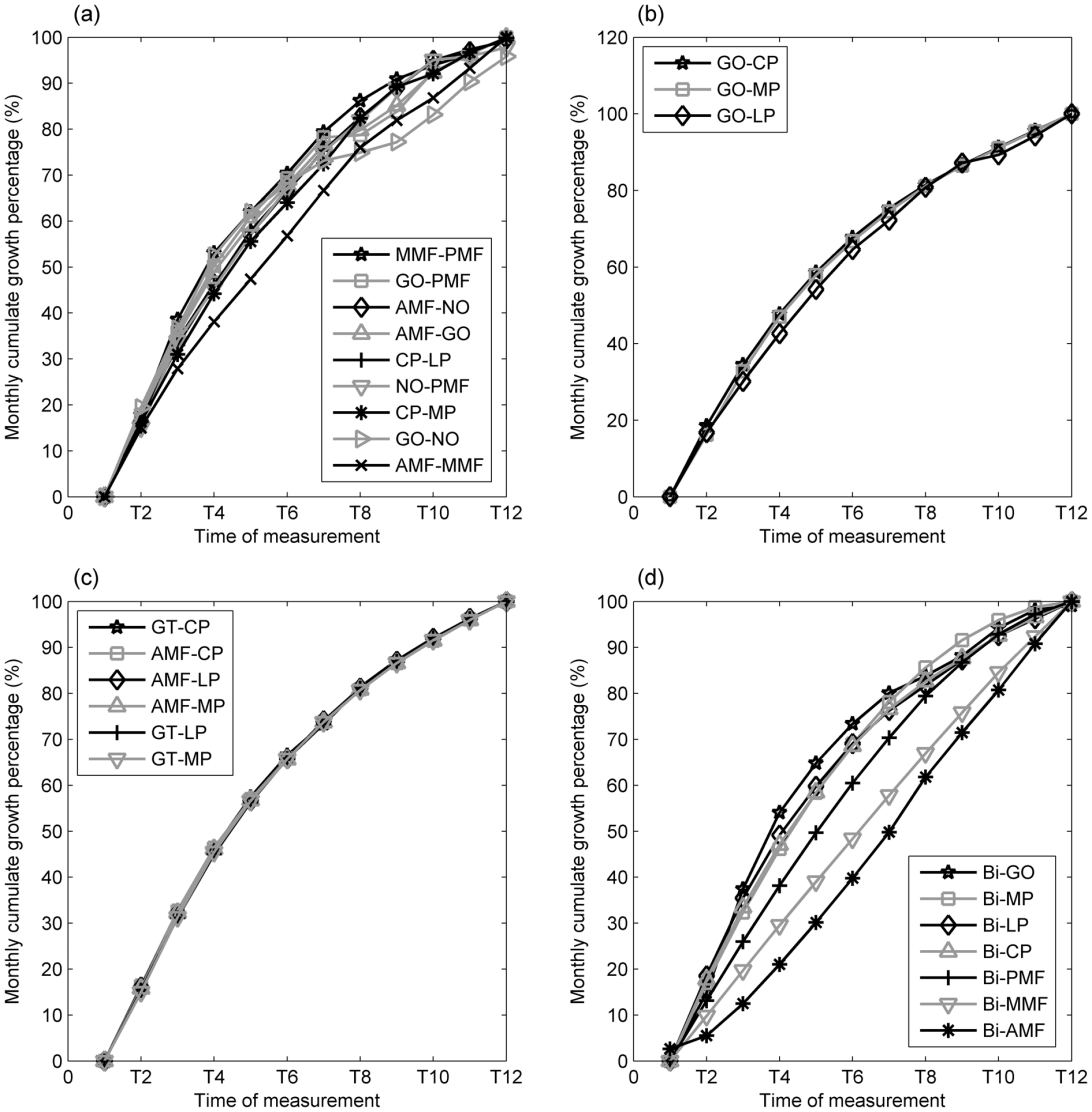
Percentages of change of inter-marker distances of the left side of the mandible over the monitoring period for the (a) anteroposterior, (b) superoinferior, (c) anteroinferior, and (d) mediolateral marker groups. Definitions of the abbreviations of the markers are given in Table.

Monthly distance changes of all the marker pairs were greatest during the first four months and then reduced over time (Fig. 5). Percentages of growth of the marker pairs appeared to be similar among the groups, reaching half of the overall growth around the 4^th^ month, except for the mediolateral group (Fig. 6). The percentage of growth of the marker pairs in the mediolateral group varied among the marker pairs, reaching half of the overall growth ranging from the 4^th^ to the 7^th^ month (Fig. 6).

**Figure 7:**
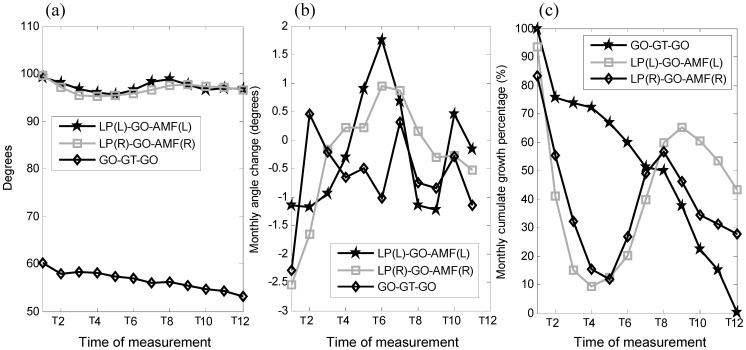
(a) Amount, (b) monthly change, and (c) percentage of change of the inter-segmental angles of bilateral LP-GO-AMF and left GO-GT-right GO. Definitions of the abbreviations of the markers are given in Table 1.

**Figure 8:**
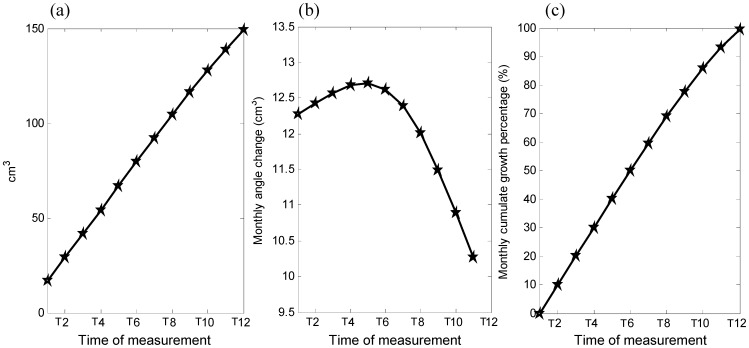
(a) Amount, (b) monthly change, and (c) percentage of change of the volume of the mandible over the monitoring period.

Among the three inter-segmental angles, LP-GO-AMF on both sides did not show much change during the growth period, but the Bi-GO-GT angle reduced gradually over time (Fig. 7). The mandibular bone volume increased over time, with a quadratic trend over the monitoring period (Fig. 8). The monthly changes of the bone volume were large during the first five months and then slowed during the last seven months (Fig. 8).

## Discussion

The current study was the first attempt in the literature to use CBCT to measure the long-term 3D morphological changes of the mandible in miniature pigs during growth. The 3D computer graphics-based reconstruction technique enabled measurement of the mandibular growth changes without having to implant metallic markers into the mandible surgically as was done in previous studies [2], [4], [5]. The current approach not only reduced the risk of damaging the growth centers of the mandible during the surgical procedure, but also reduced the psychological impact on the pigs, which may affect the growth and development of the mandible indirectly. The current technique was also free from the radiographic scattering interference from the metal implants. This approach may one day be used to monitor the growth of the human mandible when new technological developments enable imaging of the mandible at an acceptable radiation dose or free from radiation.

The growth of the mandible appeared to be anisotropic as indicated by the significantly greater mean normalized total changes of the inter-marker distances in the AP and SI groups compared with those in the AI and ML groups (Table 3). These mandible growth patterns may be related to the development of the dentition, providing necessary space for the teeth to grow upward for occlusion (growth in the SI direction) and the posterior teeth to erupt (growth in the AP direction). According to Cheng *et al*. [17], the milk dentition period of the miniature pigs occurs during the first 18 weeks, equivalent to 0.5–5 years for tooth eruption in humans; and the mixed dentition period of the pigs at 18–64 weeks was equivalent to 6–12-year old human growth. The current study measured 12 instances over the early growth of 48 weeks, corresponding to nearly 2/3 of the mixed dentition stage. The posterior segment of the mandibular body (NO-PMF) showed twice as much growth as the original size over a year, which was needed to lay the foundation of the mandibular bone for the mixed dentition stage.

The overall growth of the miniature pig's mandible over the monitoring period of 12 months was in general faster in the ramus than in the body, which was similar to that observed in human fetuses from 8 to 14 weeks [18]. In the AP group, the width of the mandible was found to occur mainly in the ramus, as indicated by the greater normalized total changes of the coronoid process and condyle (CP-LP and CP-MP) and GO-NO than the anterior part of the mandible (e.g., AMF-MMF) (Table 3). Similar findings were also found in 2D human mandibular measurements [4]. In the ML direction, the distance between the posterior parts of the mandible (Bi-GO) was found to be greater than the distance between the anterior parts (Bi-AMF, Bi-MMF, Bi-PMF). However, with the growth in the AP direction, the angle (Bi-GO-GT) was reduced gradually over time, suggesting that the increase in the ML distance of the posterior mandible was not faster than the mandibular growth in the AP direction (Fig. 7). In the ramus, the amount of growth in the SI direction (height) was greater than that in the AP direction (ramus width) (Table 3), in agreement with that observed in human fetuses [18] and New Zealand rabbits [5]. However, when the amount of growth was normalized to the original length, i.e., normalized total changes of the inter-marker distances, the opposite was found. With reference to the original inter-marker distances the growth in the AP direction (ramus width) was in fact greater than that in the SI direction (height), except for GO-MP being slightly greater than GO-NO at the end of the monitoring period (Table 3). It is thus suggested that description and prediction of the ramus growth for clinical applications should be made with reference to the original size of the bone at the initial stage of growth.

Most of the measured parameters displayed a greater amount of growth change in the initial stage of growth, as indicated by the significant rate of increase in the inter-marker distances over the first four to five months (Fig. 5), reached 50% of the total amount of growth before the fourth month, and the subsequent rate of increase slowed down over the rest of the period of the experiment (Fig. 6). The volume of the mandible also increased over time, with a rapid rate of development in the early stage of the mandibular growth up to the fifth month, followed by a reduced rate of change for the rest of the monitoring period (Fig.7). Apart from the non-linear growth patterns over time, different parts of the mandible also showed different growth patterns. For example, marker pairs in the anterior half of the mandible (AMF-MMF, MMF-PMF), and the distance between the condyle and the coronoid process (CP-LP, CP-MP), did not show significant monthly changes (Fig. 5), while significant monthly changes were found in the posterior half of the mandible body (GO-PMF) and along the SI direction of the ramus (Fig. 5). Therefore, the growth of the mandible was both non-homogeneous within the bone and non-linear over time. These phenomena may be related to the growth of teeth. It appears that the mandibular bone must increase to sufficient volume so that the teeth may erupt. Dentists and clinicians should take these phenomena into account if treatments are needed during this growth period.

In the current study, the growth of the mandible was studied in miniature pigs because of ethical considerations for human subjects, even though low-dose CBCT was used. The use of miniature pigs as an alternative to humans was supported by previous studies. Should it become safe and ethically acceptable in the future, studies on humans will be needed to confirm whether the current findings on miniature pigs indeed reflect what happens in humans. Any effect of the low radiation exposure in the current study on the mandibular growth in the pigs seems unlikely. Under the current protocol, the cumulative equivalent dose over twelve scans was estimated to be 3.156 mSv and the cumulative effective dose was 2.185 mSv, both of which were much lower than the recommended occupational dose limits for humans in a year, i.e., 20 mSv according to the 2007 International Commission on Radiological Protection (ICRP) recommendations. In addition, the ICRP concluded that for low radiation doses (i.e. <100 mSv) radiation-associated effects have not been justified [19], [20]. Furthermore, no evidence in the literature suggests that a low radiation dose such as that used in the current study will affect bone growth. Therefore, it seems unlikely that the current findings were affected by the low radiation exposure. The current study was limited to monitoring the growth of the mandible over a period of 48 weeks. This was mainly because of the limitation of the field of view of the CBCT system. With continuous growth over 48 weeks, the mandible will eventually become too large to be imaged by the CBCT. A longer period of monitoring would require a CBCT system with a larger field of view.

## Conclusions

The current study used CBCT with a computer graphics-based reconstruction technique to quantify in three dimensions the long-term growth of the mandible in miniature pigs. The growth of the mandible appeared to be anisotropic and non-homogeneous within the bone and non-linear over time. The overall growth of the miniature pig's mandible over the monitoring period of 12 months was in general faster in the ramus than in the body. These mandible growth patterns appeared to be related to the development of the dentition, providing necessary space for the teeth to grow upward for occlusion (growth in the SI direction) and for the posterior teeth to erupt (growth in the AP direction). The current results provide baseline data of the mandibular growth for future scientific and clinical applications.
