# Correction: Measurement of Mandibular Growth Using Cone-Beam Computed Tomography: A Miniature Pig Model Study

**DOI:** 10.1371/journal.pone.0132200

**Published:** 2015-07-01

**Authors:** Hsien-Shu Lin, Yunn-Jy Chen, Jia-Da Li, Tung-Wu Lu, Hau-Hung Chang, Chih-Chung Hu

There is an error in affiliation 4 for author Chih-Chung Hu. Affiliation 4 should be: Department of Mechanical Engineering, Ming Chi University of Technology, New Taipei City, Taiwan, R.O.C.
